# Effect of iron sources on the glycosylation macroheterogeneity of human recombinant IFN-γ produced by CHO cells during batch processes

**DOI:** 10.1186/1753-6561-5-S8-P114

**Published:** 2011-11-22

**Authors:** Marie-Françoise Clincke, Emmanuel Guedon, Frances T Yen, Virginie Ogier, Jean-Louis Goergen

**Affiliations:** 1 Laboratoire Réactions et Génie des Procédés UPR-CNRS 3349, ENSAIA-INPL, Nancy-Université, 54505 Vandoeuvre-lès-Nancy, France; 2 Lipidomix (EA4422), ENSAIA-INPL, Nancy-Université, 54505 Vandoeuvre-lès-Nancy, France; 3 Genclis SAS, 54505 Vandoeuvre-lès-Nancy, France

## Background

In the biopharmaceutical industry, the control of glycosylation to satisfy the quality consistency of recombinant proteins produced during a process has become an important issue. Indeed, the glycosylation pattern of recombinant proteins could be influenced by different factors including the cell line used, environmental factors such as oxygenation, temperature, shear stresses, extracellular pH... and the availability of nutrients.

As previously reported [1,2], the BDM medium is able to support a better CHO cell growth and a higher IFN-γ production compared to RPMI medium supplemented with serum. In addition, when BDM medium is used, CHO cells are capable to maintain a high percentage of doubly-glycosylated glycoforms of recombinant IFN-γ produced during the whole process. Conversely, mono-glycosylated and non-glycosylated IFN-γ forms increased during batch cultures performed with RPMI serum medium.

Iron is an important nutrient and is reported to support essential functions in cells. In this study, the impact of different iron sources on the CHO cell growth, as well as on the production and the glycosylation of a human recombinant IFN-γ were investigated.

## Materials and methods

CHO cell cultures producing human recombinant IFN-γ (CHO 320: dhfr+, α2.6 ST-) were performed in Erlenmeyer flasks (37°C, pH 7.2, 70 rpm) and in two different media, namely RPMI and BDM [3]. Whereas RPMI is a classical medium containing serum (5%), BDM is a chemically defined medium without any proteins or serum addition. BDM was supplemented with 0.1% pluronic F-68, 750 µM ethanolamine and 500 µM iron citrate.

IFN-γ was quantified using an Elisa test. Glycosylation macroheterogeneity of IFN-γ was characterized by Western Blotting (Amersham Biosciences) and each glycoform was quantified by densitometry as previously described [2].

## Results

### CHO cell growth, IFN-γ production and quality

CHO cell cultures producing human recombinant IFN-γ were cultivated in Erlenmeyer flasks, in both RPMI supplemented with 5% SVF and BDM media. Kinetics performed in BDM medium resulted in a higher maximal viable cell density and IFN-γ production compared to RPMI medium (data not shown). In fact, the higher IFN-γ production by CHO cells observed in BDM medium was mostly due to a higher cell density, since the specific rate of IFN-γ production (_q_IFN-γ) was slighly higher in the BDM medium than in the RPMI serum medium.

**Figure 1:**
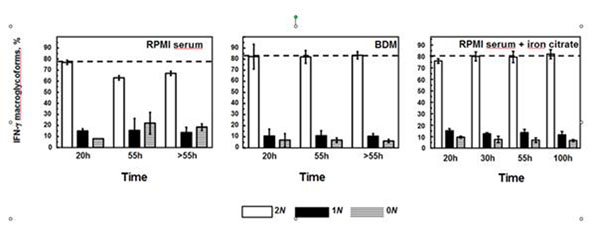
Proportion of IFN-γ macroglycoforms produced by CHO cells cultivated in various media; RPMI serum, BDM and RPMI serum supplemented with iron citrate.

In both media, three major molecular weight variants (2N, 1N, 0N) were detected during the process with a majority of IFN-γ doubly-glycosylated (2N) whatever the medium used (figure 1).

However, the quality of IFN-γ remained constant during the CHO cell cultures performed in BDM medium, contrasting with the increase in the proportion of non-glycosylated (0N) IFN-γ to the detriment of the doubly-glycosylated form (2N), during the time course of the culture of CHO cells performed in RPMI serum medium.

### Addition of iron citrate in RPMI serum strongly affects the cell growth, the production and the quality of IFN-γ

Supplementation of RPMI serum medium with iron citrate had a strong effect on kinetics of CHO cells producing IFN-γ. Indeed, a higher maximal viable CHO cell density as well as a high IFN-γ specific production rate were measured, compared to kinetics of CHO cells performed in RPMI serum medium without any supplementation (data not shown). In addition, supplementation of RPMI with iron citrate resulted in a constant glycosylation pattern of IFN-γ whereas an increase of non-glycosylated IFN-γ to the detriment of the doubly glycosylated form was observed when CHO cells are were cultivated in RPMI serum (Figure 1).

Addition of other bioavailable iron sources such as ammoniacal ferric citrate or selenium iron citrate, in RPMI serum medium resulted in the same phenomenon. However, when RPMI serum medium was supplemented with ferric-EDTA, a negative effect on the production of IFN-γ was observed (0.03 mg/10^8^ cellules vs. 0.07-0.08 mg/10^8^ cellules using iron citrate, ammoniacal ferric citrate or iron citrate complexed to selenium) despite the IFN-γ macroglycoforms was maintained constant in this condition (data not shown).

## Conclusions

Addition of iron citrate to RPMI serum medium improved cell growth, as well as IFN-γ production. Furthermore, the glycosylation pattern of IFN-γ remained constant when iron citrate was added in the medium.

Addition of ammoniacal ferric citrate, iron citrate complexed to selenium or ferric-EDTA to RPMI serum medium also allowed to maintain a constant macroglycosylation pattern of IFN-γ produced during batch cultures.

However, using ferric-EDTA in supplement of RPMI serum, the specific production rate of IFN-γ was lower compared to values obtained when other iron sources as named above were used.

Thus, the addition of a bioavailable iron source to culture media could improve the physiological cell properties as well as the quality of a recombinant protein expressed.
